# The adherence of Turkish emergency departments to geriatric guideline recommendations

**DOI:** 10.1007/s41999-024-01022-w

**Published:** 2024-07-20

**Authors:** Şimşek Çelik, Pelin Çelik

**Affiliations:** 1 Department of Emergency, Faculty of Medicine, Sivas Cumhuriyet University, Sivas, Turkey; 2 Department of Health Programs, Vocational School of Health Services, Sivas Cumhuriyet University, Sivas, Turkey

**Keywords:** Emergency department, Geriatric emergency healthcare, Geriatric emergency department guideline, Older adults

## Abstract

**Objectives:**

This study aims to determine the availability of geriatrics-friendly protocols, pieces of equipment, and physical environments alongside potential opportunities of improvement in the emergency departments of our country.

**Methods:**

This prospective, cross-sectional study was conducted between the 1^st^ and 29^th^ of February, in the year 2024, within the AI, AII, B group- and university hospitals in Turkey. A questionnaire form consisting of three subdivisions in which general pieces of information, information regarding appropriate personnel/management for geriatrics-friendly emergency departments, equipment/materials, and the presence of physical environments was presented online to the participants and the responses that were provided online were evaluated.

**Results:**

The study was conducted with a total of 175 participants from hospital emergency departments. The number of hospitals with monthly older patient application amounts of 500 and above was 133 (76.0%). It was observed that personnel/management knowledge was, relative to guideline suggestions, at low levels. When the appropriateness of personnel/management knowledge and total equipment/materials were compared to hospital roles, it was determined that university hospitals adhered closest to the guideline and that inter-group differences were significant (*p* < 0.001).

**Conclusions:**

It was determined that the compliance of emergency departments in Turkey with the criteria specified in the geriatric emergency department guidelines is at very low levels.

**Supplementary Information:**

The online version contains supplementary material available at 10.1007/s41999-024-01022-w.

## Key summary points

**Aim:**

The aim of this study was to evaluate the geriatric emergency department policies specified in the geriatric emergency department (GED) guideline in the emergency departments serving in our country, how much of the recommendations on procedures and workflow are implemented and to identify the challenges that may be encountered during the development of GEDs and to provide recommendations for their development and implementation.

**Findings:**

It has been determined that the compliance of emergency departments in Turkey with the criteria specified in the geriatric emergency department guidelines is inadequate.

**Message:**

For older patients, who make up an important part of emergency department admissions, making emergency departments suitable in terms of geriatrics-friendly protocols, equipment and physical environment is of great importance for both our country and all countries of the world in meeting the complex care needs of the older people.

**Supplementary Information:**

The online version contains supplementary material available at 10.1007/s41999-024-01022-w.

## Introduction

Older patients, constitute a rapidly growing portion of applications made to the emergency departments [1]. In our country the older people population constituted 10.2% the total population in the year 2023 who are applying to the emergency departments. As the older people population life expectancy continues to grow the number of patients aged 65 and older applying to the emergency departments will be increasing each passing day [2]. 43% of applications made to the emergency departments consists of older patients [3]. The older people often apply to the emergency departments due to multiple comorbidities, polypharmacy, older people syndromes (delirium, cognitive impairments, depression, and functional disorders) with the atypical signs and symptoms of their diseases [4, 5]. When compared to young individuals, older patients’ risk of being hospitalization rate in clinics or intensive care units is higher. Older patients require a greater number of laboratory and imaging tests have repeated emergency department visits, experience functional decline and have higher mortality rates, adding to the complexity of their care [3, 6].

There is a necessity for the environments and care procedures of the emergency departments to be made appropriate for the needs of the older people [7]. The traditional emergency care model may not be suitably equipped to meet the complicated caretaking needs of the older people [3, 8]. Few emergency departments have the training, expertise, equipment, policies and procedures needed to provide the best possible care for older patients who require more time and resources. In order to disperse these concerns, the geriatric emergency department (GED) was formed in recent years with the goal of bettering the emergency care services provided to the older people through easily-applicable approaches [3]. The common features of GED’s include interdisciplinary staff trained in geriatric emergency medicine and geriatric nursing care, evidence-based protocols for geriatric care, and physical modifications to accommodate the functional limitations of patients aged 65 years and older [8, 9]. The GED model of care has been shown to improve health outcomes, including reducing the length of stay for older patients in emergency departments and hospitals, as well as reducing emergency department admissions and readmissions [10–12]. Despite the popularisation of the GED guidelines in 2014, which have been endorsed by the American Geriatric Accreditation Criteria for Geriatric Emergency Departments (ACEP) and the American Geriatric Society, the lack of adherence to them in emergency services is striking [8, 12]. Also in 2018, the American College of Emergency Physicians (ACEP) launched the Geriatric Emergency Department Accreditation Programme, which focuses on seven areas (staffing, education, policies/protocols/guidelines/procedures, quality improvement, outcomes assessment, equipment/supplies, physical environment) to facilitate clinical care integration and distinguishes between emergency departments providing basic, developed and advanced geriatric care [13].

Due to the increased aging of the population and the need for the creation of older people friendly healthcare systems are considered, GED care models must be globally adopted into healthcare systems more extensively. The authors did not find any large-scale, nationwide studies evaluating the suitability of emergency departments for older patients according to the guidelines in the literature review. In this respect this study is significant. The aim of our study is to determine the availability of the protocols, equipment and physical environment for geriatric emergency departments specified in the guidelines in emergency departments in our country, and to provide recommendations for the development and implementation of emergency departments in the face of difficulties that may arise in the development of geriatric emergency departments.

## Materials and methods

### The type of the study

The study was conducted in a prospective, cross-sectional, and descriptive manner.

### Planning the study

There is no mandatory guideline compliance for GED in Turkey. In our study, we aimed to evaluate the current status of geriatric emergency department services in Turkey. We pragmatically preferred the Geriatric Emergency Department Accreditation (GEDA) guideline, which is regularly used in the USA and whose use in this country has been shown to be associated with better process outcomes such as length of stay, to other existing guidelines.

### The location of the study and its properties

The study was conducted between the dates February 1 2024 and February 29 2024 in the emergency departments of AI, AII, and B-group hospitals and university hospitals located in Turkey.

### The sample size of the study

The sample size of the study consisted of individuals who agreed to participate in the study who were, between February 1^st^ 2024 and February 29^th^ 2024, emergency medicine specialist physicians, faculty members of the department of emergency healthcare, or nurses responsible for the emergency department who were employed in, according to the roles assigned by ministry of health in provincial centers, central districts, and some districts; AI, AII, and B-group hospitals’ and university hospitals. The people included in our sample included academic staff (Prof. Dr., Assoc. Prof. and Assis. Prof.) and responsible emergency department nurses in university hospitals. AI group hospitals have academic staff (Prof. Dr., Assoc. Prof. and Assis. Prof.), responsible emergency medicine specialists and responsible emergency nurses. In AII and B group hospitals, there are responsible emergency medicine specialist doctors and responsible emergency nurses. AI and AII branch hospitals (Mental Health and Neurological Hospitals, Obstetrics and Pediatrics Hospitals, Oncology Hospitals, Physical Therapy and Rehabilitation Hospitals, Pulmonology Hospitals) and Oral and Dental Health Hospitals were excluded from the study. The study included a total of 221 participants. Fourteen of these consisted of data from the same hospital, 11 of the individuals had roles in the hospital which were deemed incompatible with the study, leading to their exclusion. When two or more responses were received from the same hospital, the response of the responsible academic staff of the emergency department or the responsible emergency medicine specialist was considered.

**Fig. 1:**
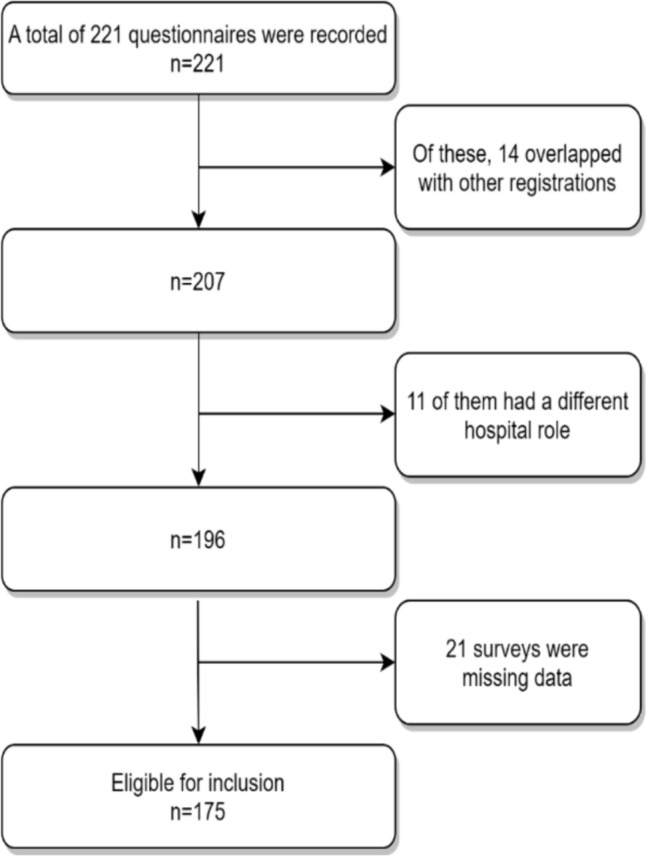
Study design

Thus, the study was conducted with 52 patients from AI, 26 from AII, 52 from B, and 35 from the university hospital, resulting in a total of 175 participants from hospital emergency departments (Fig. 1).

### The collection of data

Geriatric Emergency Department Guidelines is the product of two years of consensus-based work that included representatives from the American College of Emergency Physicians, The American Geriatrics Society, Emergency Nurses Association, and the Society for Academic Emergency Medicine [3]. For the collection of the research data, a form prepared by the researcher through the utilization of guideline information from the literature, namely “American College of Emergency Physicians Geriatric ED Accreditation Program (2023)” and “Geriatric Emergency Department Guidelines (2014)”. This form, titled “Evaluation Form Regarding the Presence of Equipment, Physical Environments, and Protocol Criteria for Optimal Geriatric Care” (Annex-1), consisted of 3 sections regarding general information, personnel/management knowledge, and equipment/material information. The 1^st^ section of the form contained questions regarding which city/institute the participants work within, their title, and the number of applications made by older patients to the emergency departments, while the 2^nd^ section consisted of 8 questions examining information such as whether or not any special flow procedures are being utilized in the emergency departments for older patients, whether or not scoring metrics are being utilized, whether or not extensive older people evaluations are conducted, whether or not scales to determine the risk of falling are utilized, emergency department workers’ status of receiving education regarding older people, and whether or not they have an emergency department medical director. The 3^rd^ section, on the other hand, consisted of 25 questions examining information regarding the presence of equipment/materials and physical environment suitable for geriatric-friendly emergency departments. These 25 questions (5 regarding materials/articles, 8 regarding equipment, 7 regarding the provision of visual orientation, 5 regarding the provision auditory orientation) The existing designs and currently utilized pieces of equipment in the emergency departments were utilized in the evaluation of their appropriateness for older patients. Participants were asked to answer questions in the questionnaire regarding the suitability of personnel/management knowledge and materials/equipment for older patients in hospitals they serve at with a “yes” if they were suitable, and a “no” if otherwise. Individuals in the hospital who met the study’s inclusion criteria were contacted by the research team through phone calls. The data collection form and informed consent forms were provided online. The responses were accepted online. In this study evaluating suitability in light of the suggestions stated by the “American College of Emergency Physicians” in the year 2014 entitled “Geriatric emergency department guidelines”, “yes” responses were evaluated as “1 point”, while “no” responses were “0 points”. The maximum attainable point from personnel/management knowledge was 8 points, and 25 points from the questions belonging to the suitability of the materials and equipment, and the suitability score was calculated as a mean value.

### Statistical analysis

All statistical analyses were conducted using the IBM SPSS Statistics 22. In addition to descriptive statistical methods (mean values, standard deviation, and frequency), Ki-square and the Kruskal Wallis test were also utilized. The statistical significance threshold was determined to be *p* < 0.05.

## Results

This study was conducted with participants from 175 hospitals’ emergency departments. The average age of the participants was 41.0 ± 8.3, with 78 of them (44.6%) being women. Most of the participants were specialist doctors, with a total number of 102 (58.3%) individuals (Table 1). The roles of the hospitals were found to be, in order of frequency, the AI group with 52 participants (29.7%), the B group with 52 (29.7%), the AII group with 36 (20.6%), and the university hospital with 35 (20.0%) (Table 1). While there was only one hospital (0.6%) with a total number of older patient applications ranging from 0 to 100, the number of hospitals with ≥ 500 were 133 (76.0%) (Table 1).

**Table 1:** Distribution of emergency department general information by region

General Information	Regions							Total *n* (%)
	MR *n* (%)	AR *n* (%)	MTR *n* (%)	CAR *n* (%)	BSR *n* (%)	EAR *n* (%)	SAR *n* (%)	
Participant title								
Prof. Dr	1 (0.6)	0 (0)	2 (1.1)	1 (0.6)	3 (1.7)	3 (1.7)	0 (0)	10 (5.7)
Assoc. Prof	7 (4.0)	2 (1.1)	1 (0.6)	5 (2.9)	1 (0.6)	1 (0.6)	1 (0.6)	18 (10.3)
Assis. Prof	4 (2.3)	4 (2.3)	5 (2.9)	5 (2.9)	2 (1.1)	1 (0.6)	1 (0.6)	22 (12.6)
Specialist Dr	15 (8.6)	13 (7.4)	11 (6.3)	15 (8.6)	17 (9.7)	14 (8.0)	17 (9.7)	102 (58.3)
Responsible Nurse	6 (3.4)	3 (1.7)	2 (1.1)	3 (1.7)	5 (2.9)	2 (1.1)	2 (1.1)	23 (13.1)
Hospital role								
AI	10 (5.7)	5 (2.9)	5 (2.9)	10 (5.7)	10 (5.7)	6 (3.4)	6 (3.4)	52 (29.7)
AII	6 (3.4)	6 (3.4)	6 (3.4)	5 (2.9)	7 (4.0)	3 (1.7)	3 (1.7)	36 (20.6)
B	8 (4.6)	5 (2.9)	7 (4.0)	8 (4.6)	8 (4.6)	7 (4.0)	9 (5.1)	52 (29.7)
University	9 (5.1)	6 (3.4)	3 (1.7)	6 (3.4)	3 (1.7)	5 (2.9)	3 (1.7)	35 (20.0)
Number of older people patients (monthly)								
0–100	1 (0.6)	0 (0)	0 (0)	0 (0)	0 (0)	0 (0)	0 (0)	1 (0.6)
101–300	1 (0.6)	0 (0)	4 (2.3)	2 (1.1)	2 (1.1)	1 (0.6)	0 (0)	10 (5.7)
301–500	7 (4.0)	0 (0)	0 (0)	8 (4.6)	6 (3.4)	7 (4.0)	3 (1.7)	31 (17.7)
≥ 500	24 (13.7)	22 (12.6)	17 (9.7)	19 (10.9)	20 (11.4)	13 (7.4)	18 (10.3)	133 (76.0)

*MR* Marmara region, *AR* Aegean region, *MTR* Mediterranean region, *CAR* Central Anatolia region, *BSR* Black Sea region, *EAR* Eastern Anatolia region, *SAR* Southeastern Anatolia region

It was determined that personnel/management knowledge was low in comparison to guideline suggestions. The number of individuals who responded “yes” to the question of whether or not their hospitals conducted evaluations to determine the waiting times of patients who were given a decision to be hospitalized was 83 (47.4%), being the highest suitability rate. On the other hand, the suitability responses given to other questions ranged from 2 (1.1%) to 28 (16.0%) individuals and there thus at low levels (Table 2).

**Table 2:** Availability of staffing/management information by region for optimal older people care in emergency departments

Personnel/management ınformation	Regions							Total n (%)
	MR *n* (%)	AR *n* (%)	MTR *n* (%)	CAR *n* (%)	BSR *n* (%)	EAR *n* (%)	SAR *n* (%)	
Is a specific workflow procedure used for older people patients?								
Yes	3 (1.7)	3 (1.7)	1 (0.6)	2 (1.1)	0 (0)	3 (1.7)	0 (0)	12 (6.8)
No	30 (17.2)	19 (10.9)	20 (11.4)	27 (15.4)	28 (16.0)	18 (10.3)	21 (12.0)	163 (93.2)
Are any risk scoring scales used?								
Yes	0 (0)	1 (0.6)	1 (0.6)	1 (0.6)	0 (0)	2 (1.1)	0 (0)	5 (2.9)
No	33 (18.9)	21 (12)	20 (11.4)	28 (16.0)	28 (16.0)	19 (10.9)	21 (12.0)	170 (97.1)
Is a comprehensive older people evaluation performed?								
Yes	4 (2.3)	1 (0.6)	1 (0.6)	6 (3.4)	6 (3.4)	4 (2.3)	6 (3.4)	28 (16.0)
No	29 (16.6)	21 (12)	20 (11.4)	23 (13.1)	22 (12.6)	17 (9.7)	15 (8.6)	147 (84.0)
Is a scale used to determine fall risk?								
Yes	1 (0.6)	2 (1.1)	2 (1.1)	2 (1.1)	1 (0.6)	4 (2.3)	1 (0.6)	13 (7.4)
No	32 (18.3)	20 (11.4)	19 (10.9)	27 (15.4)	27 (15.4)	17 (9.7)	20 (11.4)	162 (92.6)
Have emergency room staff received training in older people?								
Yes	3 (1.7)	2 (1.1)	2 (1.1)	2 (1.1)	4 (2.3)	4 (2.3)	7 (4)	24 (13.7)
No	30 (17.1)	20 (11.4)	19 (10.9)	27 (15.4)	24 (13.7)	17 (9.7)	14 (8.0)	151 (86.3)
Do you have a Geriatric Emergency Department Medical Director?								
Yes	1 (0.6)	1 (0.6)	0 (0)	0 (0)	0 (0)	0 (0)	0 (0)	2 (1.1)
No	32 (18.3)	21 (12.0)	21 (12.0)	29 (16.6)	28 (16.0)	21 (12.0)	21 (12.0)	173 (98.9)
Do you have a Geriatric Emergency Department Nursing Services Manager?								
Yes	1 (0.6)	1 (0.6)	0 (0)	0 (0)	0 (0)	0 (0)	0 (0)	2 (1.1)
No	32 (18.3)	21 (12.0)	21 (12.0)	29 (16.6)	28 (16.0)	21 (12.0)	21 (12.0)	173 (98.9)
Is an evaluation made for the waiting time in the emergency department of the patient for whom hospitalization is decided?								
Yes	14 (8.0)	10 (5.7)	9 (5.1)	16 (9.1)	13 (7.4)	11 (6.3)	10 (5.7)	83 (47.4)
No	19 (10.9)	12 (6.9)	12 (6.9)	13 (7.4)	15 (8.6)	10 (5.7)	11 (6.3)	92 (52.6)

*MR* Marmara region, *AR* Aegean region, *MTR* Mediterranean region, *CAR* Central Anatolia region, *BSR* Black Sea region, *EAR* Eastern Anatolia region, *SAR* Southeastern Anatolia region

**Fig. 2:**
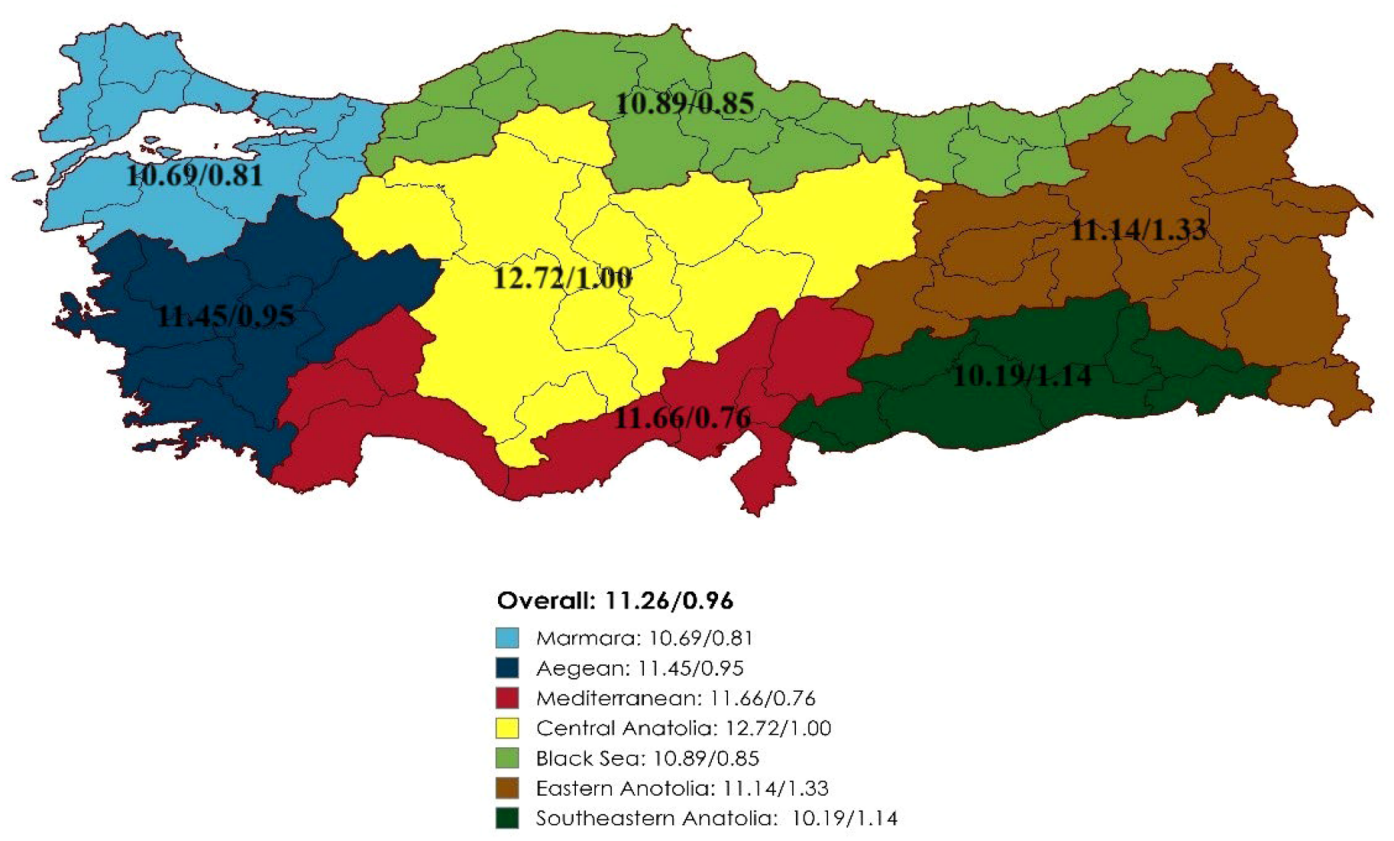
Distribution of total appropriateness scores in terms of staff-management information/emergency department equipment and supplies by region in Turkey

The average total score for all equipment and materials in Turkey is 11.26, while the average total score for staff/management information is 0.96 (Table 3, Fig. 2).

**Table 3:** Comparison of hospital roles in terms of emergency department equipment, supplies and personnel/management information

Evaluation in terms of emergency department equipment /materials and personnel/ management information	Hospital Role					
	Total	AI	AII	B	University	*p*
Furnitures						
*N*	175	52	36	52	35	0.002
Median (min.–max.)	4 (0–5)	4 (0–5)	4 (2–5)	3.5 (0–5)	5 (0–5)	
Special equipment						
*N*	175	52	36	52	35	< 0.001
Median (min.–max.)	3 (1–7)	3 (1–5)	3 (1–6)	2 (1–5)	5 (1–7)	
Visual orientation						
*N*	175	52	36	52	35	< 0.001
Median (min.–max.)	3 (0–7)	3 (0–7)	2 (1–4)	2 (1–4)	4 (0–6)	
Auditory orientation						
*N*	175	52	36	52	35	0.019
Median (min.–max.)	2 (0–4)	2 (0–4)	2 (1–4)	1 (0–3)	2 (1–4)	
Total evaluation in terms of emergency department equipment and supplies						
*N*	175	52	36	52	35	< 0.001
Median (min.–max.)	11 (3–21)	11 (5–20)	11 (7–17)	9 (5–14)	15 (3–21)	
Evaluation in terms of personnel/management information						
* N*	175	52	36	52	35	< 0.001
Median (min.–max.)	1 (0–5)	1 (0–3)	0.5 (0–4)	0 (0–1)	1 (0–5)

The score averages of the emergency departments in terms of their equipment/material and personnel/management knowledge were depicted as totals and their distributions relative to hospital roles in Table 3. The point average of the hospitals in terms of furniture was 3.62 ± 1.24, whereas a comparison between hospital roles reveals that the mean score of the university hospitals were the highest with 3.97 ± 1.54, while B group hospitals were observed to score the lowest with a mean score of 3.35 ± 0.97. This circumstance was found to be statistically significant (*p* = 0.02) (Table 3). The special equipment mean score was found to total 3.25 ± 1.39 relative to hospital roles. Special equipment score averages were at their highest in university hospitals with a value of 4.14 ± 1.70; while being the lowest in B group hospitals with a mean score of 2.57 ± 1.07. A comparison of special equipment mean scores between hospital roles reveals that the difference between the groups was statistically significant (*p* < 0.001) (Table 3).

Evaluations made relative to visual orientation equipment/materials revealed that while the mean value of the total scores was 2.73 ± 1.28, the highest value belonged to the university hospitals with 3.65 ± 1.49; whereas B group hospitals scored the lowest with a mean score of 2.15 ± 0.93. These values were significant (*p* < 0.001) (Table 3). The man values of the score totals relative to auditory orientation equipment/materials was 1.65 ± 0.83, demonstrating the highest value in university hospitals with a mean score of 1.85 ± 0.80 while demonstrating the lowest value in B group hospitals with a mean score of 1.36 ± 0.65. Through mean scores, this was found to be the lowest value. It was determined that the conclusions were significant (*p* = 0.019) (Table 3). While the mean scores of all equipment and materials (furniture, special equipment, visual and auditory equipment/materials) was found to be 11.26 ± 3.32, this value was at its highest in university hospitals with 13.62 ± 4.69, and its lowest in B group hospitals with a mean value of 9.48 ± 2.16. The difference between the groups in terms of hospital roles was found to be statistically significant (*p* < 0.001) (Table 3). Comparisons made in terms of personnel/management knowledge yielded a mean total score of 0.96 ± 1.14, where the highest value of 1.88 ± 1.45 was demonstrated by university hospitals and the lowest value of 0.19 ± 0.39 was demonstrated by B group hospitals. The difference between the groups was significant (p < 0.001) (Table 3).

## Discussion

In this study, the suitability of the emergency departments in Turkey to the geriatric emergency departments specified in the guideline was evaluated. In the majority of the hospitals included in the study (76.0%), it was determined that the monthly number of older patient admissions was 500 or more. The literature data, in line with our study, has also reported that the number of older people patients applying to the emergency departments is gradually increasing [1, 3, 6]. The traditional emergency department care model remains insufficient in fulfilling the complex care needs of older people adults [8]. For this reason, it is very important to make appropriate geriatric emergency department planning for older patients whose population and emergency department admission rates are increasing day by day.

We found that our employee/management information scores and geriatrics education given to emergency department staff were low compared to the recommended levels set out in the guidelines. It was determined in our study that the geriatrics education provided to emergency department workers was at low levels relative to guideline suggestions. Devriandt et al. reported in their study that geriatrics education provided in Belgium’s emergency departments was insufficient (approximately 2,9 h per year) [4]. Another study, conducted in Belgium, found that compliance with standard guidelines for the care of older patients in emergency departments varied (15.6–78.1%), and the proportion of participants who thought the guidelines were applicable ranged from 9.4% to 50.0% [6].

August 2023 According to the GED accreditation criteria, it was emphasised that after the decision to hospitalised patients in level 1 and 2 geriatric emergency departments, the length of stay in the emergency department should be monitored while waiting to be transferred to the inpatient unit [14]. In our study, it was determined that approximately half of patients who were hospitalized had their hospitalization duration monitored as they waited for a transfer to a patient unit and that hospitalization duration monitoring was higher than our other personnel/management data.

In this study, it was found that emergency departments only demonstrated a value higher than half of the maximum suitability score in terms of geriatric-friendly equipment/materials for furniture, while other values were lower than half of the maximum attainable suitability score. A study conducted in Belgium in recent years demonstrated that pressure ulcer-reducing beds (84.4%), heating blankets (81.3%), bed heights (87.5%), bedside nightstands (81.6%) were suitable to the standards at large rates, whereas hearing devices (6.3%), preservative catheters (38.7%), and walking-assistance devices (6.3%) were below standard suitability. It was determined that the compliance rates of physical environment criteria with the standards were between 6.3% and 84.4%. In the same study, some criteria were met to a very high degree, while others had very low levels of compliance. [6]. While some criteria (auditory orientation criteria) were at low levels of suitability levels in our study, the overall suitability score in terms of emergency department equipment/materials was found to be approximately half of the maximum score and was considered as positive.

Delirium is a common and potentially fatal cause of emergency department admissions in older patients [15]. Therefore reducing the risk of delirium in the emergency department is very important. It has been demonstrated that lighting improvements such as soft lights and exposure to natural lights as well as auditory improvements such as special rooms and acoustically developed curtains are beneficial in the reduction of delirium [3]. Special areas and structural improvements (flooring, beds, special equipment, etc.), designed in consultation with experts in the field of geriatrics, allow older adults to move more freely and reduce the risk of delirium and similar situations [16]. In the light of these data, we believe that the risk of delirium will be reduced if emergency departments are transformed into appropriate geriatric care centres according to the guideline in terms of equipment/materials.

When we compared the compliance of all participating EDs with the guidelines’ criteria for plans and equipment according to hospital type, we found that university hospitals were more compliant, while Group B hospitals were less compliant. Since university hospitals are the last centre where patient referrals are made and the centre where all comorbid patients, including older patients, are treated, university hospitals have a higher awareness of this issue. In addition, universities have more established management protocols, so these recommendations may have been met without specific attention to geriatric care.

Despite the popularization of the 2014 Geriatric Emergency Department Guidelines, geriatric emergency patient care services continue to demonstrate differences and the aforementioned guidelines are not applied in most emergency departments [17, 18]. No framework exists for prioritization; the best or fundamental applications are not known [19]. For hospitals included in the study, in a vast majority of cases, the formation of special GED units due to their older patient applications being high would be significant in providing more effective and high-quality treatments and caretaking services to older patients. In addition to special areas and equipment, specialists such as geriatrics doctors, geriatrics nurses, social service specialists, physiotherapists, palliative medicine advisors, and pharmacists must be present. Geriatric Emergency Department care can be provided in all institutions, from universities to small community hospitals [8, 14]. It may not be possible for the emergency departments participating in the study in our country to provide 24-h the team and the environment related to the issues specified in the guidelines due to the inadequacy of the existing health personnel in today’s conditions. Triage and diagnosis can be difficult in older patients with multiple comorbidities, polypharmacy and functional–cognitive impairment, who often present with clinical signs and symptoms of acute illness. However, the use of geriatric emergency department interventions, starting with pilot projects in EDs with lower rates of geriatric presentation, may help to address both structural and process of care challenges related to the special care needs of older patients. Moreover, all older people individuals may not require extensive geriatric evaluations or special equipment. Focusing on the target group of 10–12% and providing them with extensive geriatric care would, in the first stage, be sufficient [8].

This study contains various limitations. First, our data only covers public hospitals, and does not contain data on private hospitals. Second, as self-report information surveys were utilized, absent and over reporting were permitted, meaning that results may be biased. Third, approximately 10% (21) of the responses we received were excluded due to missing data. This may have limited our findings. Finally, the survey only investigated areas of the “American College of Emergency Physicians Geriatric ED Accreditation Program (2023)” and the Geriatric Emergency Department Guidelines (2014) guideline suggestions that were directly linked to patient caretaking. To conclude, areas regarding quality improvement and conclusion measurements were not investigated. This path was chosen to ensure that the survey remained as short as possible. These areas we were unable to measure also play an indispensable role in the emergency departments provided for older patient caretaking. Thus, future studies must aim to additionally evaluate these areas.

## Conclusion

Older people require specially designed emergency healthcare services. Guideline-adherent emergency departments were found to be at insufficient levels in Turkey for older patients.

## Supplementary Information

Below is the link to the electronic supplementary material.

Supplementary file1 (DOCX 18 KB)

## Data Availability

Study data were provided by the corresponding.
