# Supplementary material for: The adherence of Turkish emergency departments to geriatric guideline recommendations
Source: Eur Geriatr Med. 2024 Jul 20;15(5):1267–75. doi: 10.1007/s41999-024-01022-w (PMC11615048; doi:10.1007/s41999-024-01022-w)
Supplement: Supplementary file 1 — Supplementary file1 (DOCX 18 KB) [file 41999_2024_1022_MOESM1_ESM.docx]

**Assessment Form on the Availability of Equipment, Physical Environment and Protocol Criteria for Optimal Geriatric Care in Emergency Departments**

**(ANNEX-1)**

| **Part 1** | |
| --- | --- |
| Age |  |
| Gender |  |
| City/institution where you work |  |
| Your Title |  |
| Monthly number of geriatric patients admitted to your institution |  |
| **Part II (Personnel/management information)** |  |
| 1) Is a specific workflow procedure used for geriatric patients? | Yes ( ) No ( ) |
| 2) Are any risk scoring scales used? | Yes ( ) No ( ) |
| 3) Is a comprehensive geriatric evaluation performed? | Yes ( ) No ( ) |
| 4) Is a scale used to determine fall risk? | Yes ( ) No ( ) |
| 5) Have emergency room staff received training in geriatrics? | Yes ( ) No ( ) |
| 6) Do you have a Geriatric Emergency Department Medical Director? | Yes ( ) No ( ) |
| 7) Do you have a Geriatric Emergency Department Nursing Services Manager? | Yes ( ) No ( ) |
| 8) Is an evaluation made for the waiting time in the emergency department of the patient for whom hospitalization is decided? | Yes ( ) No ( ) |
| **Part III (Information on the availability of equipment/materials and physical environment)** | |
| **A)** **Furnitures** | |
| 1) Are there reclining chairs suitable for patient transfer and examination? | Yes ( ) No ( ) |
| 2) Are there sturdy armrests on the furniture? | Yes ( ) No ( ) |
| 3) Are the beds/stretchers at a level where elderly patients can easily stand up? | Yes ( ) No ( ) |
| 4) Are there extra thick/soft stretcher beds to prevent the development of decubitis? | Yes ( ) No ( ) |
| 5) Are the upholstery made of soft, easy-to-wipe, non-porous and seamless material? | Yes ( ) No ( ) |
| **B) Special Equipment** | |
| 1) Are there body heating devices and heating blankets? | Yes ( ) No ( ) |
| 2) Are there liquid heaters? | Yes ( ) No ( ) |
| 3) Do you have non-slip mats/matting? | Yes ( ) No ( ) |
| 4) Do they have bedside tables? | Yes ( ) No ( ) |
| 5) Are there walking aids? | Yes ( ) No ( ) |
| 6) Do you have hearing aids? | Yes ( ) No ( ) |
| 7) Is there respiratory equipment including fiberoptic intubation tools? | Yes ( ) No ( ) |
| 8) Are there urinary catheter supplies, including condom catheters? | Yes ( ) No ( ) |
| **C) Visual Orientation** | |
| 1) Is there a large-faced analog clock in every patient room? | Yes ( ) No ( ) |
| 2) Can lighting be provided with natural light (daylight)? | Yes ( ) No ( ) |
| 3) Are there high quality signage and wayfinding signs? | Yes ( ) No ( ) |
| 4) Are the sockets designed to allow patients to turn the lighting in their area on and off at will? | Yes ( ) No ( ) |
| 5) Are the walls matte and light colored? | Yes ( ) No ( ) |
| 6) Are contrasting colors used between horizontal and vertical surfaces? | Yes ( ) No ( ) |
| 7) Is there a matt floor covering on the floor? | Yes ( ) No ( ) |
| **D) Auditory Orientation** | |
| 1) Are there sound-isolating curtains/paravans between patient stretchers? | Yes ( ) No ( ) |
| 2) Are there individually designed patient rooms? | Yes ( ) No ( ) |
| 3) Are alarm sounds from high noise sources such as monitors reduced? | Yes ( ) No ( ) |
| 4) Can patients be provided with a space where they can listen to music or watch a television program without disturbing others? | Yes ( ) No ( ) |
| 5) Are relatives encouraged to meet with their relatives in cases of prolonged stay in the emergency department? | Yes ( ) No ( ) |
